# UNDERSTANDING THE UNIQUE NEEDS OF VULNERABLE OLDER ADULTS IN A COMMUNITY-BASED TELEHEALTH PROGRAM

**DOI:** 10.1093/geroni/igac059.1003

**Published:** 2022-12-20

**Authors:** Melody Schiaffino, Zhan Zhang, Pratik Chaudhari, Jina Huh-Yoo

**Affiliations:** San Diego State University, San Diego, California, United States; CSIS, New York, New York, United States; CSIS, New York, New York, United States; College of Computing and Informatiics, Philadelphia, Pennsylvania, United States

## Abstract

Vulnerable older adults benefit from community-based telehealth programs (CTP) that facilitate remote health monitoring with support from trained personnel. This study assessed acceptability with such technology as a self-reported measure of comfort among participants in an on-going CTP, the Telehealth Intervention Program for Seniors (TIPS). We analyzed data from participants across 20 sites (N=2279), 38% responded to their comfort with technology (n=866). We modeled self-reported factors to explore the association with technology acceptability. There was more comfort with technology than not (53.5% vs 46.5%). Participants under age 65, those reporting better vs poorer health (p<0.0001) and a happier mood state (p<0.0001) were more likely to be comfortable. Older adults and much older adults reported greater odds of comfort compared with those under 65. Better health status was associated with 84.5% greater odds of acceptability compared to those with poor (AOR 1.85; 95CI 1.28-2.65). Happier participants reported 56% greater odds of comfort compared with those reporting unhappiness. Though only marginally significant, non-English speaking participants reported greater odds of comfort compared to English proficient. While ethnicity was not associated, our marginal significance for language suggests a need to continue exploring. Our work demonstrates the need to address the unique needs of older adults.

